# ProNGF/NGF Modulates Autophagy and Apoptosis through PI3K/Akt/mTOR and ERK Signaling Pathways following Cerebral Ischemia-Reperfusion in Rats

**DOI:** 10.1155/2022/6098191

**Published:** 2022-03-29

**Authors:** Yanbo Li, Fengbo Wu, Muke Zhou, Jie Zhou, Shuhui Cui, Jian Guo, Junhao Wu, Li He

**Affiliations:** ^1^Department of Neurology, West China Hospital of Sichuan University, Chengdu 610041, China; ^2^Department of Otolaryngology, Head and Neck Surgery, West China Hospital, Sichuan University, Chengdu 610041, China

## Abstract

NGF is involved in the process of autophagy; however, the underlying mechanisms of proNGF/NGF on autophagy in cerebral ischemia-reperfusion (CIR) remain unclear. This study explored the potential pathway of proNGF/NGF in mediating autophagy and apoptosis and thereby contributed to poststroke neurological rehabilitation. In this study, PC12 cell lines and male SD rats were used to simulate CIR; it was found that within 24 h reperfusion, proNGF was the predominant form of *Ngf* while after 24 h NGF was produced by proNGF transformation. The mature NGF was found to protect neurons against autophagic and apoptotic damage caused by CIR, but proNGF can cause both autophagic and apoptotic neuronal damage. The protective effect of NGF is associated with the activation of the PI3K/Akt/mTOR and ERK pathway and, as well as the change of autophagy-related proteins. On the other hand, proNGF promoted the ERK pathway increasing autophagy and affected the apoptosis-related proteins *in vivo* and *in vitro*. These results were also verified in male SD rats with CIR that neurological deficit caused by CIR can be rescued by recombinant and wild-type NGF, and vice-versa by proNGF.

## 1. Introduction

Cerebral ischemia-reperfusion (CIR) is a significant pathophysiological process in cerebral ischemic infarction for the salvage of acutely injured brain tissue. In the clinical setting, many poststroke patients who do receive the reflow therapy in time during the acute stage still have a significant long-term disability and a residual motor deficit was found in more than two-thirds of the survivors [1–3]. Autophagy, an evolutionarily conserved process delivering damaged organelles and long-lived proteins to the lysosome system for clearance has been identified as a key regulator in cerebral ischemia [4–8], along with apoptosis in the maintenance and development of the cellular function [9]. Based on data from recent variant research, this quality control mechanism might be viewed as a “double-edged sword” in cerebral ischemia [10, 11]. A series of signaling pathways including protein kinase activation is necessary for the process [8, 12–14], such as the mTOR signaling pathway, MAPK signaling pathway, and HIF-1*α* signaling pathway [15]. Since there is no effective targeted therapy on these cascades for poststroke rehabilitation, research on autophagy-related molecules for clinical use is still in its initial stages and further research has been going on. Growing evidence constantly suggested that the nerve growth factor (NGF) participated in the homeostatic cell processes, markedly in autophagy and apoptosis, and is known to be essential to neuronal survival in the central and peripheral neural systems. In the physical state of the rat brain, the firstly discovered [16] mature form of NGF was experimentally detected to affect some autophagic proteins and thus to result in autophagic activation or dysregulation [17, 18]. It was also demonstrated to play an important role in CIR, promoting neurons' proliferation and survival [19–21] by interacting with multiple ligands including TrkA, sortilin, and p75^NTR^ [21–26]. As such a promising neurotrophic factor, NGF had been clinically tried to apply into other neuropathies, Alzheimer's disease (AD) therapy [27] and neuropathic pain control [28], by injecting the combined AAV-NGF vector and using targeted anti-NGF-antibodies, but vector mistargeting and blood-brain barrier defense were observed as the defects by clinical trials [29]. New approaches to NGF therapy for central nervous diseases are necessary. The precursor of NGF (namely, proNGF) is another best characterized protein that was then demonstrated to be the predominant form of NGF in the mature rodent central nervous system (CNS) under many pathophysiological states [30] (including the CIR process [26]). The current research has suggested that proteins of mature NGF and proNGF have a delicate balance while coexisting in general and they may reciprocally interfere with each other [31]. To have a better understanding of the two proteins may provide new strategies for NGF therapy. proNGF, in the up-to-date researches, was separately investigated for its sole role after CIR and was found to be long-term upregulated [26], and otherwise, it could bind to sortilin [32] and p75^NTR^ [33] in the induction of apoptosis. This evidence demonstrated that any of the two NGF products may impact neural regeneration and neurological rehabilitation of the CNS in CIR and share comparable neurotrophin activity. There may be more details unveiled about the proNGF/NGF signaling in the process of CIR. Since mature NGF had been observed to mediate Akt/mTOR inhibition of autophagic flow activation in myocardial ischemia-reperfusion [34], the function of mature NGF and proNGF after CIR may potentially correlate with this kind of signaling.

Thus, it was postulated that NGF could affect the neurological function resulting from cerebral ischemia by modulating Akt/mTOR to mediate apoptotic and autophagic activities after CIR, in the form of mature NGF and proNGF. The levels of mature NGF- and proNGF-mediating neuronal death, signaling cascades, and the activation of autophagic flow, as well as apoptosis, were examined, thereby developing a novel therapeutic strategy to improve the neurological function, both *in vivo* and *in vitro*. This research indicated a new role of NGF as a signal to the autophagic cell death after CIR and provides new thoughts to affect the balance of mature NGF and proNGF as the potential therapeutic strategy.

## 2. Results

### 2.1. OGD/R Induces a Dynamic Change of Protective NGF and Harmful proNGF to PC12 Cells

**Figure 1:**
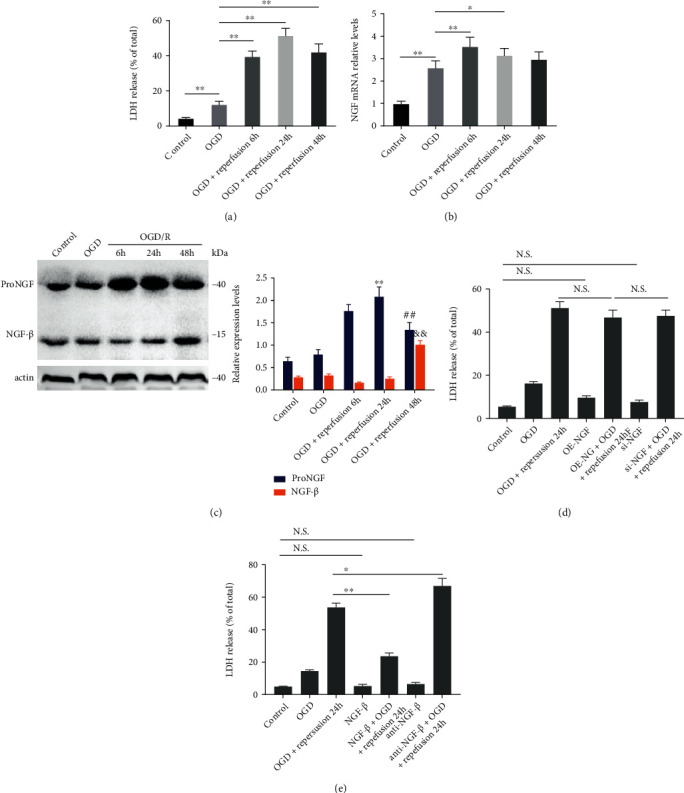
Mature NGF and proNGF's dynamic change in the OGD/R model. Mature NGF and proNGF were detected in different reperfusion groups as follows: control cells, cells exposed to OGD without reperfusion, cells exposed to OGD/R 6 h, cells exposed to OGD/R 24 h, and cells exposed to OGD/R 48 h. Dynamic change of the NGF mRNA relative level and LDH release after OGD/R at different time points; ^∗^*P* < 0.05 and ^∗∗^*P* < 0.01 (a, b). Western blots of proNGF and NGF-*β* after OGD/R at different time points. ^∗∗^*P* < 0.01 vs control, ^##^*P* < 0.01 vs OGD/R 24 h, and ^&&^*P* < 0.01 vs OGD/R 24 h (c). The viability of PC12 cells was measured by LDH release in seven groups as follows: control cells, cells exposed to OGD, cells exposed to OGD/R 24 h, cells exposed to OE-NGF, cells exposed to OE-NGF + OGD/R 24 h, cells exposed to si-NGF, and cells exposed to si-NGF + OGD/R 24 h. The effect of exogenous NGF-*β*/inhibitor and genic interference on PC12 cells after OGD/R. ^∗^*P* < 0.05, ^∗∗^*P* < 0.01; N.S.: no significance (d, e).

The PC12 cell line was utilized as the *in vitro* model of cerebral ischemia, and LDH release was detected in different reperfusion conditions to evaluate cell viability to examine the neurotrophic activity of NGF and proNGF on cells following OGD/R. After reperfusion of 24 h, a peak of LDH release was observed, and then, it dropped slightly at 48 h. The mRNA expression of the NGF gene was also tested in the abovementioned time points, and it was found to have the same trend with the peak at OGD/6 h reperfusion. ProNGF expressed to the peak after OGD/24 h reperfusion and gradually decreased after 48 h reperfusion, whereas NGF-*β* expression decreased significantly after OGD/reperfusion and the downward trend began to slowly pick up at 48 h after the reperfusion. These findings confirmed NGF's protective role and proNGF's harmful role, and proNGF may transfer to NGF 24 hours after OGD/R (as shown in Figures 1(a)–1(c)).

### 2.2. Exogenous NGF-*β*/Inhibitor Affects the Viability of PC12 Cells after OGD/R While Interference of NGF Expression Does Not

As OGD/24 h reperfusion was an important turning point, OGD/24 h reperfusion was chosen as the main model and the viability of the cells was observed under the NGF intervention from the genic and molecular levels. Different groups of PC12 cells were exposed to OGD/24 h reperfusion with OE-NGF (overexpression NGF) plasmid, si-NGF (NGF with siRNA), recombinant 2.5S NGF-*β* protein, or anti-NGF-*β* antibody treatment. The results showed that genic overexpression or silencing of NGF had no significant effect on the LDH release of PC12 cells compared with the nontreatment group. The administration of recombinant 2.5S NGF-*β* protein could decrease the release rate of LDH induced by OGD/R significantly, and anti-NGF-*β* antibody could cancel this change (as shown in Figures 1(d) and 1(e)).

### 2.3. Recombinant NGF-*β* Protein Decreases the Levels of Autophagy and Apoptosis While proNGF Increases Them in a Model of 24 h Oxygen-Deprivation Reperfusion Injury in PC12 Cells

**Figure 2:**
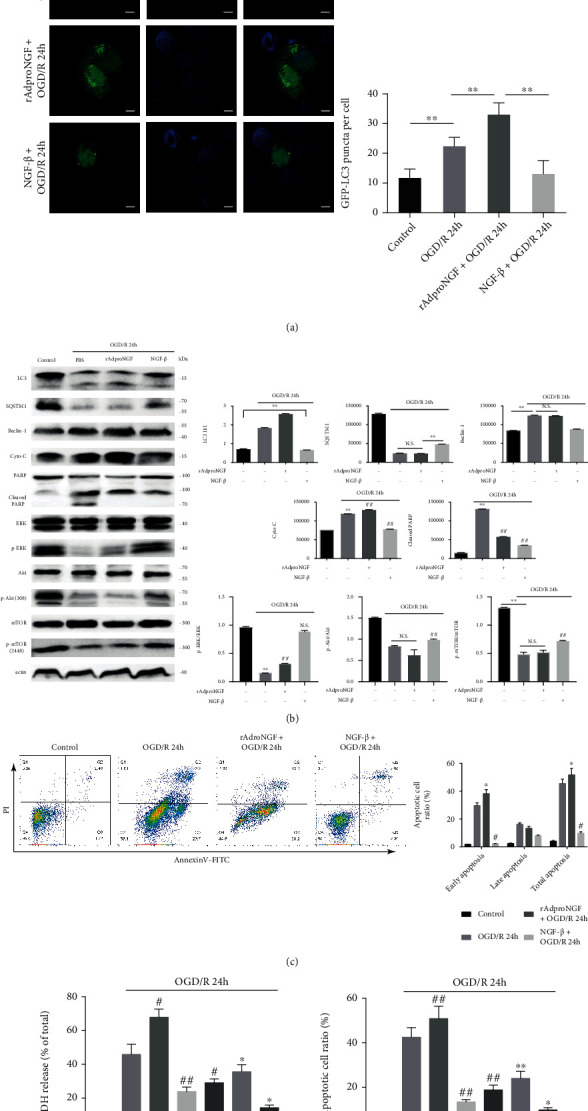
Effect of rAdproNGF and recombinant NGF-*β* protein on autophagy and apoptosis of OGD/R-exposed PC12 cells. The four groups studied are as follows: control cells, cells exposed to OGD/R 24 h, cells exposed to rAdproNGF + OGD/R 24 h, and cells exposed to NGF-*β* + OGD/R 24 h. PC12 cells were pretransfected with GFP-LC3 plasmid and treated with rAdproNGF and recombinant NGF-*β*, respectively. The fluorescent staining of LC3 was observed among the groups; ^∗∗^*P* < 0.01 (a); bar = 10 *μ*m. LC3, SQSTM1, Beclin-1, Cyto-C, PARP, ERK, Akt, and mTOR were detected by Western blots. ^∗^*P* < 0.05 and ^∗∗^*P* < 0.01 vs the control group; ^#^*P* < 0.05 and ^##^*P* < 0.01 vs OGD/R 24 h; N.S.: no significance (b). LC3, SQSTM1, Beclin-1, Cyto-C, PARP, ERK, Akt, and mTOR were detected by Western blots. ^∗∗^*P* < 0.01 vs the control group; ^##^*P* < 0.01 vs OGD/R 24 h; N.S.: no significance (c). Annexin V-FITC was tested for the apoptosis; ^∗^*P* < 0.05 vs the OGD/R 24h group; Viability of cells was observed for rAdproNGF and recombinant NGF-*β* protein when exposed to autophagic inhibitor 3-MA.^#^*P* < 0.05 vs the rAdproNGF + OGD/R 24 h group. ^∗^*P* < 0.05 and ^∗∗^*P* < 0.01 vs the no 3-MA group; ^#^*P* < 0.05 and ^##^*P* < 0.01 vs OGD/R 24 h (d, e).

To further investigate the effect of proNGF and NGF-*β* on autophagy and apoptosis in OGD/R, rAdproNGF or recombinant NGF-*β* protein was added into PC12 cells. Since GFP-LC3 is a substrate for autophagic degradation, it is regarded to be a marker for autophagic levels in mammalian cells. The effect of all neurotrophic factors on this established autophagic reporter was also assessed. The results showed that LC3 fluorescence in normal PC12 cells increased when induced by OGD/R 24 h. rAdproNGF exacerbated OGD/R-induced LC3 change while the NGF-*β* group relieved it (Figure 2(a)). The ratio of LC3-II : LC3-I, a determinant indicating increased autophagic cargo processing, was further established by quantification of immunoblotted cell lysates on LC3 conversion, increased under the action of rAdproNGF, and attenuated when NGF-*β* was added. In sorted PC12 cells, the expression of beclin-1, an essential autophagy inducer, increased and SQSTM1 (p62/sequestosome 1), a specific autophagic substrate, decreased after OGD/R (as shown in Figure 2(b)). NGF-*β* inhibited the modifications of the two molecules that could identify and sequester autophagic cargo. rAdproNGF did not alter the level of beclin-1 and SQSTM1 significantly. These results indicated that OGD/R 24 h increased the autophagy level of PC12 cells and rAdproNGF elevated the autophagy activity while NGF-*β* reduced it.

Meanwhile, to understand the contemporaneous apoptosis level in PC12 cells during the OGD/R, PI-Annexin V-FITC dual labeling was conducted by flow cytometry (FCM) analysis. It was observed that OGD/R 24 h induced the apoptosis in normal PC12 cells especially early apoptosis. rAdproNGF worsened the reperfusion injury by sharply increasing the early apoptosis with compensation for the slightly decreased late apoptosis, resulting in elevated total apoptosis. In contrast, the treatment of NGF-*β* significantly reduced both early and late apoptosis (as shown in Figure 2(c)). Apoptosis was also evaluated using Cyto-C, an indicator of incomplete mitochondrial integrity, and cleaved PARP, a marker of apoptosis. After OGD/R, the expression of Cyto-C and cleaved PARP increased in PC12 cells, indicating that the process increased apoptosis in PC12 cells. When compared with the OGD/R group, rAdproNGF treatment led to an increase in Cyto-C and NGF-*β* had the opposite result, which meant that there was an increase in apoptosis under the action of rAdproNGF and the opposite under NGF-*β*. However, the expression of cleaved PARP decreased after the administration of rAdproNGF and NGF-*β*, and compared with rAdproNGF, NGF-*β* resulted in a lower expression of cleaved PARP indicating that NGF-*β* was more potent to make PARP cleaved in the process of apoptosis.

Hence, it can be concluded that rAdproNGF may significantly increase the level of apoptosis and autophagy in PC12 cells after OGD/R 24 h, while recombinant NGF-*β* protein reduces them.

### 2.4. *Ngf* Regulates Cell Autophagy and Apoptosis via PI3K/Akt/mTOR and ERK Signaling in a PC12 Cell Model of Ischemia-Reperfusion Injury

Cell autophagy and apoptosis are known to be associated with many signaling, such as PI3K/Akt, mTOR, and ERK signaling. It was checked whether NGF-regulated autophagy and apoptosis were partially mediated by these pathways. ERK, a well-known signaling molecule, had decreased phosphorylation after OGD/R. However, the treatment of rAdproNGF and NGF-*β* could restore ERK phosphorylation modification caused by OGD/R. Besides, the classic pathway molecules of Akt and mTOR (mammalian target of rapamycin) were also tested for their role in the process. OGD/R could reduce Akt and mTOR phosphorylation. NGF-*β* could upregulate the phosphorylation of Akt and mTOR. But the expression of p-Akt and p-mTOR was similar after the administration of rAdproNGF (Figure 2(b)).

The early autophagy inhibitor 3-MA was used to determine whether proNGF/NGF is PI3K dependent to affect PC12 cell survival in the process of OGD/R. The results showed that OGD/R significantly increased LDH release and apoptosis in cells and rAdproNGF aggravated this cell damage while NGF-*β* attenuated it. When treated with 3-MA, the OGD/R-induced LDH release and apoptosis in the rAdproNGF group were restored while the two indicators in the NGF-*β* group were further attenuated (see Figures 2(d) and 2(e)). These data demonstrated that proNGF/NGF regulated cell survival PI3K-dependent and via PI3K/Akt/mTOR and ERK signaling. Moreover, 3-MA treatment was found to restore part of LDH release and apoptosis, suggesting that autophagy could harm cell viability in the process of OGD/R 24 h.

### 2.5. NGF/proNGF Activates NF-*κ*B/NLRP3 Signaling in a PC12 Cell Model of Ischemia-Reperfusion Injury

**Figure 3:**
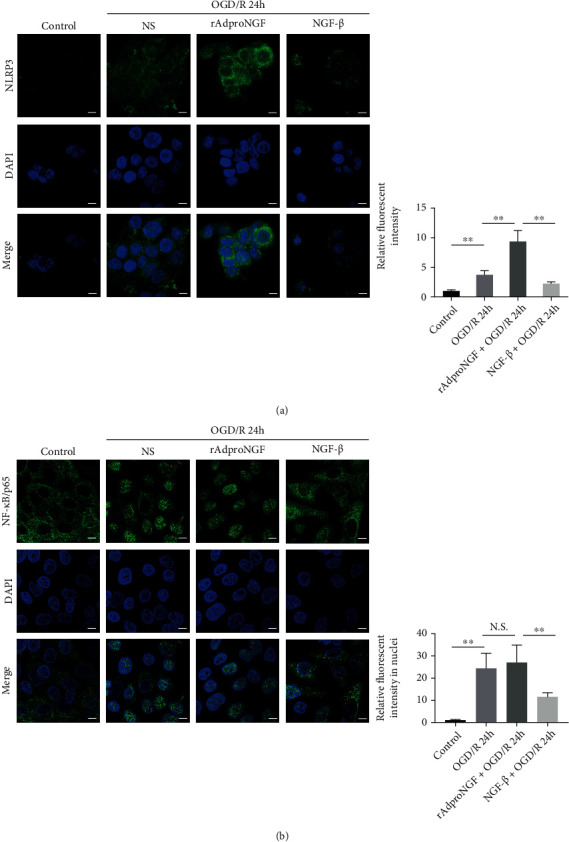
NF-*κ*B/NLRP3 signaling was activated by rAdproNGF and NGF-*β*. The four groups studied are as follows: control cells, cells exposed to OGD/R 24 h + normal saline (NS), cells exposed to rAdproNGF + OGD/R 24 h, and cells exposed to NGF-*β* + OGD/R 24 h. NLRP3 and NF-*κ*B were detected by immunofluorescence; ^∗∗^*P* < 0.01; N.S.: no significance (a, b); bar = 10 *μ*m.

Tests were conducted on NLRP3-related signaling on the PC12 cell line in the model of OGD/R. It was observed that OGD/R induced the upregulation of NF-*κ*B nuclear activation and NLRP3, and rAdproNGF could further increase NF-*κ*B and NLRP3 activity, while NGF-*β* impaired this effect induced by OGD/R (as shown in Figure 3).

### 2.6. Neurological Deficit and Cerebral Infarction Can Be Rescued by Recombinant NGF-*β* Protein and Wild-Type proNGF (rWdAdproNGF), Whereas Mutant-Type proNGF (rAdproNGF) Cannot in a Rat Model of Ischemia-Reperfusion Injury

**Figure 4:**
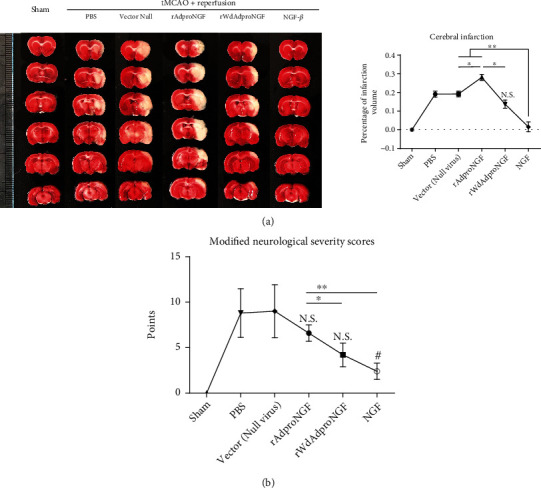
Percentage of infarction volume was calculated to visually judge the effect of NGF on cerebral ischemic injury. ^∗^*P* < 0.05; ^∗∗^*P* < 0.01; N.S.: no significance vs the vector group (a). mNSS evaluation reflects a protective role of NGF-*β* and rWdAdproNGF and an evil role of rAdproNGF. ^#^*P* < 0.05 and N.S. = no significance vs the vector group; ^∗^*P* < 0.05; ^∗∗^*P* < 0.01 (b).

Rats with transient middle cerebral artery occlusion and reperfusion (tMCAO/R) were used as the *in vivo* model of ischemic stroke. For this model, rats were subjected to tMCAO/R with different treatments of the recombined mutant-type or wild-type proNGF vector and exogenous NGF-*β*. For indications of MCAO injury, the neurological function deficit and the volume of the visual infarct were assessed. NGF-*β* significantly shrank the infarction volume compared with that of the nontreatment group. On the contrary, the rAdproNGF group had a larger infarction volume which was statistically different from the rWdAdproNGF and NGF-*β* treatment groups. The effect of wild-type proNGF treatment was in the midst (Figure 4(a)). The neurological function on tMCAO/R rats was assessed by conducting an mNSS score (*N* = 8/group). The mNSS score was a common scale reflecting the comprehensive neurological severity after the cerebral ischemia process. A significant therapeutic efficacy of NGF-*β* could be found in tMCAO/R rats while an unobvious effect of rAdproNGF was observed, compared with that in the nontreatment group. However, rWdAdproNGF showed a trend of a relatively larger protective effect than rAdproNGF as shown in Figure 4(b).

### 2.7. Autophagy and Apoptosis Are Strengthened by proNGF Overexpression While Weakened by Recombinant NGF-*β* Protein and Wild-Type proNGF, through Affecting the Akt/mTOR Signaling Cascade, in a Rat Model of Ischemia-Reperfusion Injury

**Figure 5:**
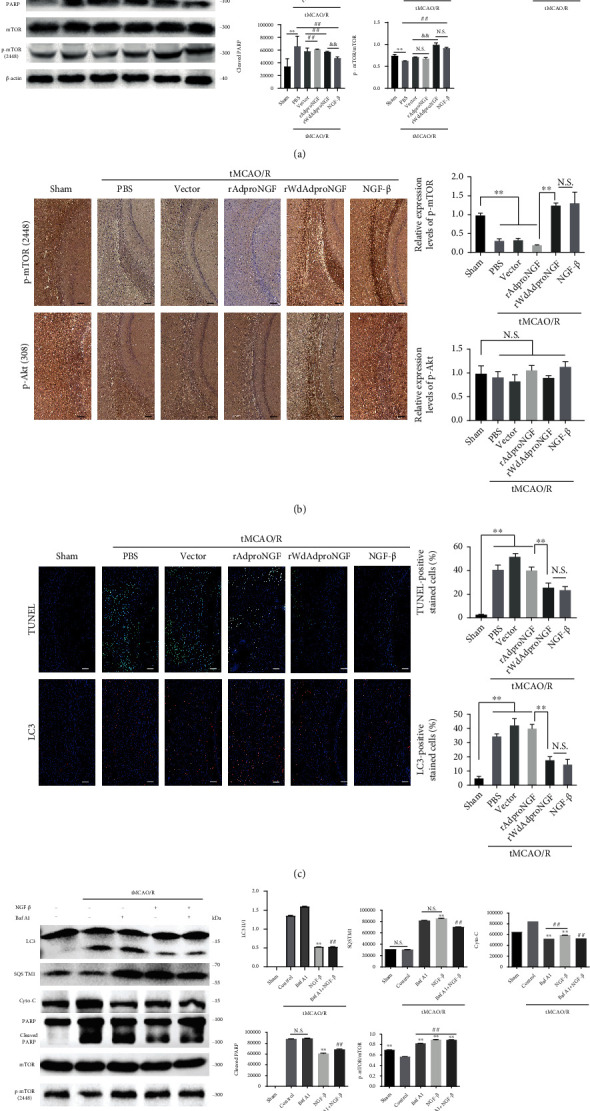
NGF regulated autophagy and apoptosis through Akt/mTOR signaling in a rat model of ischemia-reperfusion injury. Six groups were divided: sham group, PBS group, vector (null virus) group, rAdproNGF group, rWdAdproNGF group, and NGF-*β* group. LC3, SQSTM1, Cyto-C, PARP, ERK, Akt, mTOR, and TUNEL were detected by immunoblotting, immunohistochemistry, and immunofluorescence in control and rAdproNGF-, rWdAdproNGF-, and NGF-*β*-treated MCAO models. ^#^*P* < 0.05; ^&^*P* < 0.05; ^∗∗^*P* < 0.01; ^##^*P* < 0.01; ^&&^*P* < 0.01; N.S.: no significance (a–c); bar = 100 *μ*m. Baf-A1 was added into NGF-*β* therapy. Quantification of immunoblotted proteins in brain extracts was conducted for LC3, SQSTM1, Cyto-C, PARP, and mTOR. ^∗∗^*P* < 0.01 vs the tMCAO/R group; ^##^*P* < 0.01 vs the nontreatment group; N.S.: no significance (d).

Since the two surgeries caused side effects to rats, 48 h after tMCAO/R was chosen as the observing timepoint. In an animal model, it was confirmed that the induction of ischemia-reperfusion could augment the level of autophagy and apoptosis at 48 h with increased LC3, SQSTM1, Cyto-C, cleaved PARP, and TUNEL immunofluorescence. The treatment of rWdAdproNGF and NGF-*β* was observed to result in a weakened TUNEL, decreased LC3 II transformation, and increased SQSTM1 to restore the autophagy level induced by ischemia-reperfusion and to make Cyto-C and cleaved PARP decrease as with apoptosis, whereas rAdproNGF was not observed to have the same statistical change on neuronal cells *in vivo* as it did on PC12 cells *in vitro* in which the levels of LC3, Cyto-C, p-mTOR, and SQSTM1 were similar to those of the nontreatment group. Cleaved PARP increased slightly with a more increased PARP. The TUNEL immunofluorescence was not significantlychanged after rAdproNGF treatment compared with that of the vector group. In addition, Akt and mTOR were also assessed for verification *in vivo*. rWdAdproNGF and NGF-*β* could also be observed to affect Akt/mTOR signaling *in vivo* by increasing p-Akt and p-mTOR in rat brain tissue after ischemia-reperfusion. In contrast to the vector group, the rAdproNGF group showed similar p-Akt and p-mTOR, as well as similar autophagy and apoptosis. The findings showed that NGF may influence autophagy via the Akt/mTOR signaling pathway. Autophagy regulator BafA1, a V-ATPase inhibitor blocking the fusion of autophagosomes, was also added to the medium of a different group to observe the changes of the indicators. It was found that NGF-*β* could mimic the action of BafA1 to inhibit autophagy and reduce autophagy. Consequently, NGF-*β* may act as a blocker downstream of autophagic flux. Moreover, in the process of CIR, apart from autophagy, NGF-*β* could also protect the neurons from apoptotic injury (shown in Figure 5).

## 3. Discussion

Reperfusion injury is a prevalent phenomenon in patients with ischemic infarction. Reperfusion causes significant neuron death, leading to a variety of neurological deficits [35]. NGF had been demonstrated to regulate the remaining pyramidal neuron function in CIR from different reports [19, 21]. This is the first study on the relationship between the two characterized protein products of NGF and OGD/R. The mechanism of modulation of autophagy and apoptosis under the action of proNGF/NGF in the process of CIR was explored. It was found that within 24 h ischemia reperfusion, proNGF was the predominant form of *Ngf*. Mature NGF could coexist with the precursor and play a protective role in the process, inhibiting harmful autophagy and apoptosis. ProNGF/NGF regulated autophagy and apoptosis mainly through PI3K/Akt/mTOR and ERK signaling.

First, the dynamic change of NGF function following OGD/R was investigated and the results presented a curve of how proNGF/NGF altered after 24 h of OGD/R process. That is, proNGF may cause cell injury in the first 24 hours and subsequently transfer into its mature form, which has a protective role at 24–48 hours after OGD/R. On basis of this, we used OE-NGF and siRNA to interfere with the expression of *Ngf*. Interestingly, cell viability hardly changed under transcriptional intervention; however, exogenous NGF-*β* could protect the cells from OGD/R injury. This was considered to result from the simultaneous change of proNGF and NGF-*β*, the protein possessed by proNGF, to make the effects of the two neurotrophins cancel each other out. Furthermore, additional investigations have revealed that the prodomain can form transient intramolecular contacts with the mature NGF domain and that these contacts might have heterogeneous conformational changes [36]. This NGF loop conformation promotes various targeted recognition and thus affects NGF expression [37], which is another possible interpretation of the mechanism of proNGF/NGF change in this study. Furthermore, in exogenous treatment, the amount of LDH release was much less when treated by NGF-*β* than by anti-NGF-*β* antibody, which might be correlated with the receptor binding rate and may also be related to the ratio of proNGF/NGF. Arisi et al. have suggested that a certain ratio of proNGF/NGF-*β* can induce a specific response, upregulating or downregulating some genes whose expression is different from that of a single proNGF or NGF and ligands [38]. Tiveron et al. also found that the imbalance of proNGF/NGF-*β* levels can induce learning, memory deficits, neurodegenerative alterations, and spontaneous epileptic discharges in transgenic mice [39]. From this evidences, it was postulated that in CIR, there probably existed a proNGF/NGF-*β* balance affecting the poststroke injury. Rodent models of cerebral ischemia also showed that different ratios of the two proteins were directly associated with the neurological function deficits induced by MCAO. Otherwise, mature NGF has been continuously tested for its use in neurological diseases and made good progress; however, in the central neural system, NGF therapy still confronted the big challenge of vector mistargeting and blood-brain barrier delivery. Aiming for a delicate balance between mature NGF and proNGF may be a promising approach to CNS disease therapy.

In this study, autophagy which is an alternative method to acquire energy for cell survival after ischemia [40] was predicted to be a process leading to cell injury. The level of early-stage apoptotic cells in PC12 cells after OGD/R was significantly increased in the rAdproNGF group, but decreased in the NGF-*β* group. This directly suggests that these two proteins may have opposite regulatory receptors and pathways in the apoptosis and autophagy processes, which is consistent with the proapoptotic effects of proNGF and p75^NTR^ and sortilin ligands and the neuroprotective effects of NGF and TrkA, as former studies suggested. According to the current data, mature NGF phosphorylates the downstream PI3K/Akt/mTOR pathway, restoring enhanced autophagic flux and apoptotic activity. rAdproNGF, on the other hand, may affect cellular autophagy by increasing ERK phosphorylation. PI3K/Akt and ERK were used to be demonstrated as classic pathways associated with cellular proliferation, differentiation, and survival. mTOR had also been proved to be the downstream effector of the PI3K/Akt pathway. These findings demonstrated that mature NGF and proNGF are involved in autophagy and apoptosis, even possibly in inflammasome activation, via the PI3K/Akt/mTOR and ERK signaling pathways, respectively. Furthermore, it was found that rAdproNGF still augmented the level of apoptosis; this result may be caused by other pathways or by the interruption of proNGF/NGF balance when administered rAdproNGF treatment.

Neurological function deficits were assessed *in vivo* for mechanism verification using the visual insult size and neurobehavioral tests. In the tMCAO/R model, we used NGF-*β*, rWdAdproNGF, and rAdproNGF which could not transfer into NGF, to give a poststroke therapy. The analyses on infarction volume and mNSS showed that NGF-*β* significantly improved CIR injury while rWdAdproNGF had the trend to moderately improve the neurological function. On the other hand, rAdproNGF did not affect them too much. Previous studies had demonstrated that NGF-*β* was a protective neurotrophic factor after ischemia-reperfusion [25, 41–43], similar to this study. rAdproNGF did not have the same impact *in vivo* as it did *in vitro*, which might be related to the presence of endogenous NGF products. The addition of rAdproNGF may disrupt the original balance of proNGF/NGF *in vivo* and offset part of the neuroprotective effect that NGF should give and restore the Akt/mTOR signaling that should be activated. The three intervention groups presented different environments of proNGF/NGF coexistence, based on which the net effect of the NGF family in CIR was distinct. The results of the *in vivo* model indirectly support the aforementioned conjecture of the important conversion ratio of proNGF and NGF. NLRP3 and NF-*κ*B were also tested *in vitro* with different proNGF/mature NGF balance, and the results showed a significant correlation between the NF-*κ*B-NLRP3 pathway and Ngf. This may give us a new insight into the mechanism of *Ngf*'s impact on CIR.

**Figure 6:**
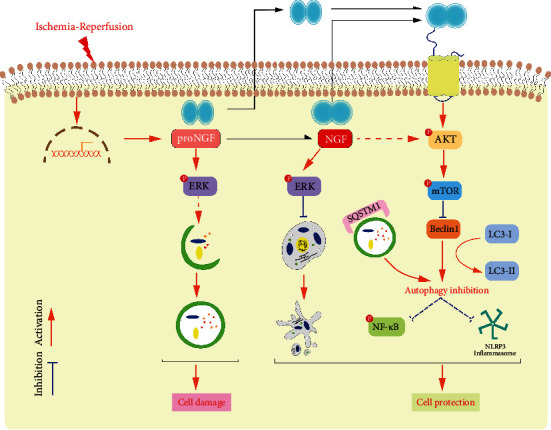
The proposal mechanisms of proNGF/NGF in cerebral ischemia-reperfusion.

Our study mainly focused on the *Ngf* gene's work in CIR by modulating autophagic and apoptotic signaling pathways. Further research is needed to determine if and how the signal cascade impacts the downstream effector, such as autophagy-lysosomes or another route. However, this study gave important information that proNGF/NGF had its dominant pathway in influencing cell survival and modulating the balance of proNGF/NGF could indirectly have an impact on the cell signaling. Besides, this study was the initial work for the proNGF/NGF balance in the disease model of CIR; the ratio of proNGF/NGF was not discussed deeply. The proposed mechanisms of proNGF/NGF in CIR injury were depicted in Figure 6. More experiments and verifications are required to achieve a goal for CIR therapy by modulating the two major neurotrophic molecules.

In conclusion, in the regulation of OGD/R by the Ngf gene, the predominant protein substance within 24 hours was proNGF. There may be a dynamic coexistence state of proNGF/NGF during 24 and 48 hours after reperfusion. After OGD/R, the expression of autophagy and apoptosis increased, while autophagy appeared to evoke cell death and injury. In this process, proNGF induced autophagy and apoptosis, while recombinant NGF-*β* could effectively inhibit autophagy and apoptosis caused by reperfusion. The expression products of the Ngf gene regulated apoptosis and autophagy mainly through the PI3K/Akt/mTOR and ERK pathways. There may be a crosslink between autophagy and apoptotic pathways. The similar trend could be observed in the motor behavioral test and cerebral infarction volume of rats.

## 4. Materials and Methods

### 4.1. Cell Culture and Viability Assay

Rat pheochromocytoma-derived PC12 cells (obtained from SIBS, China) are a common model to establish neuron-like cells and can be used as a potential target for neurotrophic effects. They were grown in a complete medium that comprised of RPMI 1640 Medium, 10% fetal bovine serum, and 5% heat-inactivated horse serum, in a humidified atmosphere of 5% CO_2_ at 37°C. Following the published protocol, PC12 cells were mechanically dislodged by forceful aspiration and replanted into multiwell culture dishes with poly-L-lysine coated at 120000 cells/dish [44]. We determined the extent of apoptosis of PC12 cells by using the Annexin V/PI staining flow cytometry method according to the manufacturer's protocol. We also used the Pierce LDH Cytotoxicity Detection Kit (Thermo Scientific) to quantitatively assess LDH activity in culture medium based on the kit protocol to detect the cell death induced by OGD/reoxygenation.

### 4.2. Vector Packaging

For overexpression NGF, the coding regions of NGF were amplified from rat cDNA by real-time quantitative reverse transcription-polymerase chain reaction (RT-qPCR) using gene-specific primers. The products, on one hand, were used to construct an overexpression NGF plasmid and, on the other hand, were cloned into the ADV. Furin sequencing was blocked as needed when constructing furin-resistant proNGF ADV. NGF-specific recombinant adenovirus (rAdNGF), proNGF-specific furin-resistant recombinant adenovirus (rAdproNGF), wild proNGF-specific recombinant adenovirus (rWdAdproNGF), and empty carrier recombinant adenovirus were packaged at Shanghai GeneChem Co. Ltd. For NGF knockdown, a selected siRNA sequence for rat NGF was induced into a plasmid. The resultant si-NGF plasmid in this study was purchased from RiboBio Co. GFP-LC3 reporter constructs were used to measure LC3 levels visually. Shanghai GeneChem Co. Ltd. packaged the corresponding viral vector plasmid.

### 4.3. OGD/R (Oxygen and Glucose Deprivation/Reperfusion) Model

The OGD/R model was initiated by removing the original cell culture medium and washing the cells twice with glucose-free medium (glucose-free RPMI 1640 Medium, 1% fetal bovine serum, and 2% heat-inactivated horse serum) in an oxygen-free incubator (95% N_2_ and 5% CO_2_) for 6 h (OGD). Following the OGD procedure, glucose was reintroduced by substituting the glucose-free medium with the original medium after two PBS washes and cells were cultured for an additional 18 hours (OGD-R) under normal conditions. PC12 cells that were just concurrently washed by glucose-free RPMI 1640 Medium twice without OGD served as controls. siRNA, rAdproNGF, 2.5S NGF-*β*, and anti-NGF antibody were added into the medium before OGD/R and other detections.

### 4.4. Animals

Young adult male Sprague-Dawley rats (weight 290 ± 20 g) were housed in standard 12-hour light/dark cycles at a regulated temperature and moisture. The food and water were freely accessible. If the animals were in good condition, they were housed in pathogen-free cages with solid floors covered with sawdust for one week before undergoing surgeries. Before the surgery, the animals had to fast for 12 hours. During the light cycles, all surgeries and experimental procedures were performed. All animal experiments were conducted according to the ethical regulations set by the local animal welfare committee as well as the National Institutes of Health guide for the care and use of laboratory animals (NIH Publications no. 8023, revised 1978).

#### 4.4.1. Constitution of MCAO (Middle Cerebral Artery Occlusion)/Reperfusion Model

Male Sprague-Dawley rats (290 ± 20 g) were randomly chosen for the reversible MCAO model to mimic cerebral ischemia-reperfusion. They were anesthetized with 2% halothane in a gas mixture of 60% N_2_O and 40% O_2_ using a face mask, and the blood flow into the MCA (middle cerebral artery) was blocked with an intraluminal suture under the operating microscope as previously demonstrated [45]. The right common carotid artery (CCA), external carotid artery (ECA), and internal carotid artery (ICA) were revealed briefly. A monofilament nylon suture, determined by the weight of the animal (suture with a diameter of 0.43 ± 0.03 mm for rats weighing 270–280 g; 0.47 ± 0.03 mm for rats weighing 300–310 g), was advanced from the ECA into the lumen of the ICA until it blocked the origin of the right middle cerebral artery (MCA). Animals were reanesthetized with halothane one hour after the blockage, and reperfusion was accomplished by slowly withdrawing the suture. Zea-Longa evaluation was used to screen out the eligible rats with a score of 1 to 3, which were then randomized into six groups for brain stereotaxic injection. Rats with a Zea-Longa score of 0, subarachnoid hemorrhage, or death within 24 h were excluded, and the complementary rat MCAO/R models were established to meet the insufficiency.

### 4.5. Brain Stereotaxic Injection

One day after the MCAO/R surgery, animals were anesthetized with isoflurane as previously described and fixed in the stereotaxic apparatus. A longitudinal incision was made in the middle of the head, and a small hole was drilled through the skull on the ipsilateral side of the infarction. A 10 *μ*l microsyringe (Hamilton Company, USA) was used to slowly puncture to the predefined position. Bregma was used as stereotaxic zero, and the stereotaxic coordinates used for intraventricular injections were −0.92 mm Bregma, 1.55 mm lateral, and 3.75 mm from the skull surface. The incision was sutured after the operations. Experimental groups consisted of the following (*N* = 8/group): (1) PBS group: MCAO/R rats treated with PBS; (2) vector (null virus) group: MCAO/R rats treated with empty carrier recombinant adenovirus; (3) rAdproNGF group: MCAO/R rats treated with 2 × 10^10^ PFU/ml rAdproNGF; (4) rWdAdproNGF group: MCAO/R rats treated with 1.5 × 10^10^ PFU/ml rWdAdproNGF; and (5) NGF-*β* group: MCAO/R rats treated with 100 *μ*g/ml NGF-*β*.

### 4.6. Behavioral Tests

Behavioral tests were started the day before the operation as a baseline and then performed on day 2 after tMCAO/R or sham surgery. Modified neurological severity scores (mNSS) were used to evaluate the neurological function of the animal, grading on a scale from 0 to 18 (normal score, 0; maximal deficit score, 18) [46], as shown in Table 1. It is a comprehensive test for motor, sensory, reflex, and balance. The higher the total score, the more severe the neurological function was.

### 4.7. Tissue Preparation

One day after surgeries, rats were euthanized and the brains were further tested. Rats were deeply anesthetized as previously described and the brains were quickly taken out after skull gaffing for fixation, TTC staining, or Western blotting. The brains were fixed for one week with fresh cold 10% neutral formalin before sectioning on a Leica CM1900 Cryostat (Leica Instruments, Germany) at 30 *μ*m thickness. The sagittal section of a 6 mm thick brain tissue block through the rostro-caudal axis of the brain was collected at 3 mm from the frontal pole and then stored for immunohistochemistry and immunofluorescence. The fresh brain was used for TTC staining. The Western blotting specimens were quickly frozen in liquid nitrogen.

### 4.8. Infarct Volume

Serial coronal sections (+4.7 mm to −5.5 mm/Bregma) [47] were soaked in 2% 2,3,5-triphenyltetrazolium chloride (TTC) phosphate buffer at 37°C for 30 min in the dark. Normal brain tissues were stained red, while infarct tissues were not stained (white). The sections were soaked in 4% paraformaldehyde phosphate buffer for 30 min, arranged in order, and scanned. The ratios of infarct regions to total brain areas were computed to measure the infarct volume.

### 4.9. Western Blotting

PC12 cells that were collected on the ice were homogenized in a lysis buffer. PC12 cell samples were centrifuged at 12000 rpm for 5 min at low temperature to remove cellular debris and concentrate. Brains were ground in liquid nitrogen before homogenization and centrifugation in the same manner as PC12 cells. The total protein concentration in the retentate from the brain or PC12 cells was determined by BCA Assay (Beyotime Biotechnology). Proteins were separated in SDS-PAGE, transferred to PVDF, and blocked for 1 h at room temperature in TBS-T with skim milk. Membranes were separately incubated with the following specific primary antibodies at 4°C overnight: rabbit polyclonal anti-LC3 (Abcam, UK, use a concentration of 2 *μ*g/ml), rabbit monoclonal anti-SQSTM1 (Abcam, UK, 1 : 1000), rabbit monoclonal anti-Beclin 1 (Abcam, UK, 1 : 1000), rabbit monoclonal anti-Cyto C (Abcam, UK, 1 : 5000), rabbit monoclonal anti-PARP (Abcam, UK, 1 : 1000), rabbit monoclonal anti-cleaved PARP (Abcam, UK, 1 : 2000), rabbit polyclonal Akt antibody (CST, USA, 1 : 1000), rabbit polyclonal phospho-Akt antibody (CST, USA, 1 : 1000), rabbit polyclonal ERK1/2 antibody (CST, USA, 1 : 1000), rabbit monoclonal phospho-ERK1/2 antibody (CST, USA, 1 : 1000), rabbit polyclonal anti-mTOR (Abcam, UK, 1 : 2000), or rabbit polyclonal phospho-mTOR antibody (Abcam, UK, 1 : 1000), and secondary antibodies. For detection, protein electrophoresis bands were photographed after electrochemiluminescence reaction (Ultra High Sensitivity ECL Kit, MCE). Image-Pro Plus 6.0 software was used to analyze the OD of objective protein photographs.

### 4.10. Immunohistochemistry and Immunofluorescent Assays

Brain tissue with the range of +2.76 mm to −3.24 mm/Bregma was stained from each brain, and 3 random nonoverlapping fields were assessed from each section by a blinded observer using Image-Pro Plus 6.0 software. For immunohistochemical analysis for p-Akt, p-mTOR of the brain tissue was performed on representative serial paraffin-embedded sections as previously described. They were stained with rabbit polyclonal phospho-Akt antibody (CST, USA, 1 : 100) or rabbit polyclonal phospho-mTOR antibody (CST, USA, use a concentration of 5 *μ*g/ml) followed by anti-antibody of HRP-conjugated goat anti-rabbit IgG H&L (1 : 7500 in TBS-T, Abcam, UK). Standard fluorescent immunohistochemistry procedures and the primary and secondary antibodies indicated below were used to treat the brain tissue for immunofluorescence: rabbit polyclonal anti-LC3 antibodies (Abcam, UK, use a concentration of 1 *μ*g/ml) and HRP-conjugated goat anti-rabbit IgG H&L (1 : 7500 in TBS-T, Abcam, USA). PC12 cells were fixed by formaldehyde and blocked by Donkey serum and then were processed with standard immunofluorescent techniques using primary and secondary antibodies of goat polyclonal to NLRP3 (Abcam, UK, use a concentration of 10 *μ*g/ml), rabbit monoclonal to NF-*κ*B p65 (Abcam, UK, 1 : 100), and secondary antibody. The nuclear area was defined according to DAPI staining.

### 4.11. Other Reagents and Antibodies

3-Methyladenine (3-MA) and 2,3,5-triphenyltetrazolium chloride (TTC) were obtained from Sigma-Aldrich (St. Louis, MO, USA). Bafilomycin A1 (Baf-A1) was obtained from Selleck. The primary antibody that targeted towards *β*-actin and terminal deoxynucleotidyl transferase-mediated fluorescein-dUTP nick end labeling (TUNEL) assay kit were purchased from the ProteinTech Group Inc. (Wuhan, China). The horseradish peroxidase- (HRP-) conjugated secondary antibodies for Western Blotting were obtained from Cell Signaling Technology (Boston, USA).

## Figures and Tables

**Table 1:** Modified neurological severity score point.

Motor tests	
Raising rat by tail	3
Flexion of the forelimb	1
Flexion of the hindlimb	1
Head moved >10°to the vertical axis within 30 s	1
Placing the rat on the floor (normal = 0; maximum = 3)	3
Normal walk	0
Inability to walk straight	1
Circling toward the paretic side	2
Falls down to the paretic side	3
Sensory tests	2
Placing test (visual and tactile tests)	1
Proprioceptive test (deep sensation, pushing the paw against the table edge to stimulate limb muscles)	1
Beam balance tests (normal = 0; maximum = 6)	
Balances with steady posture	0
Grasps the side of the beam	1
Hugs the beam and 1 limb falls down from the beam	2
Hugs the beam and 2 limbs fall down from the beam, or spins on the beam (>60 s)	3
Attempts to balance on the beam but falls off (>40 s)	4
Attempts to balance on the beam but falls off (>20 s)	5
Falls off; no attempt to balance or hang on to the beam (<20 s)	6
Reflex absence and abnormal movements	4
Pinna reflex (head shake when the auditory meatus is touched)	1
Corneal reflex (eye blink when the cornea is lightly touched with cotton)	1
Startle reflex (motor response to a brief noise from snapping a clipboard paper)	1
Seizures, myoclonus, myodystony	1
Maximum points	18

One point is awarded for inability to perform the tasks or for the lack of a tested reflex: 13–18: severe injury; 7–12: moderate injury; 1–6: mild injury.

## Data Availability

The data that support the findings of this study are available from the corresponding author upon reasonable request.

## Supplementary Materials

Supplementary Materials The changes on MAP2 and ProNGF/NGF at different time intervals after OGD/R *in vitro* and *in vivo* were shown in figure S1.
