# Supplementary material for: ProNGF/NGF Modulates Autophagy and Apoptosis through PI3K/Akt/mTOR and ERK Signaling Pathways following Cerebral Ischemia-Reperfusion in Rats
Source: Oxid Med Cell Longev. 2022 Mar 29;2022:6098191. doi: 10.1155/2022/6098191 (PMC8983267; doi:10.1155/2022/6098191)
Supplement: Supplementary Materials — The changes on MAP2 and ProNGF/NGF at different time intervals after OGD/R in vitro and in vivo were shown in figure S1. [file 6098191.f1.docx]

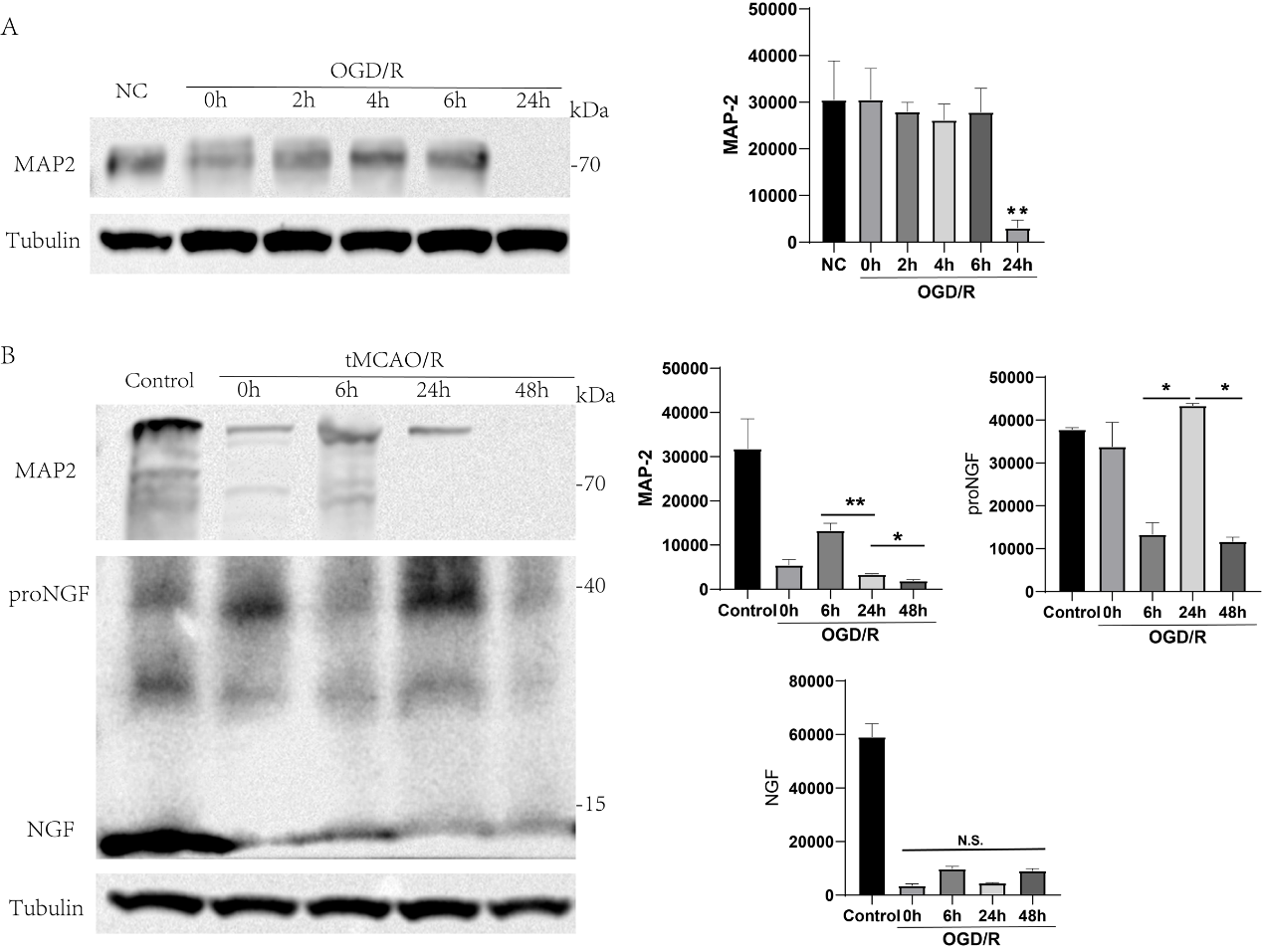


Figure S1. The changes on MAP2 and ProNGF/NGF at different time intervals after OGD/R in PC12 cells (A) or tMCAO/R in rats (B).
